# Metabolism in stem cell–driven leukemia: parallels between hematopoiesis and immunity

**DOI:** 10.1182/blood.2022018258

**Published:** 2023-01-14

**Authors:** Kevin M. Rattigan, Martha M. Zarou, G. Vignir Helgason

**Affiliations:** 1 Wolfson Wohl Cancer Research Centre, School of Cancer Sciences, University of Glasgow, Glasgow, United Kingdom

## Abstract

Our understanding of cancer metabolism spans from its role in cellular energetics and supplying the building blocks necessary for proliferation, to maintaining cellular redox and regulating the cellular epigenome and transcriptome. Cancer metabolism, once thought to be solely driven by upregulated glycolysis, is now known to comprise multiple pathways with great plasticity in response to extrinsic challenges. Furthermore, cancer cells can modify their surrounding niche during disease initiation, maintenance, and metastasis, thereby contributing to therapy resistance. Leukemia is a paradigm model of stem cell–driven cancer. In this study, we review how leukemia remodels the niche and rewires its metabolism, with particular attention paid to therapy-resistant stem cells. Specifically, we aim to give a global, nonexhaustive overview of key metabolic pathways. By contrasting the metabolic rewiring required by myeloid–leukemic stem cells with that required for hematopoiesis and immune cell function, we highlight the metabolic features they share. This is a critical consideration when contemplating anticancer metabolic inhibitor options, especially in the context of anticancer immune therapies. Finally, we examine pathways that have not been studied in leukemia but are critical in solid cancers in the context of metastasis and interaction with new niches. These studies also offer detailed mechanisms that are yet to be investigated in leukemia. Given that cancer (and normal) cells can meet their energy requirements by not only upregulating metabolic pathways but also utilizing systemically available substrates, we aim to inform how interlinked these metabolic pathways are, both within leukemic cells and between cancer cells and their niche.

## Visual Abstract

**Figure:**
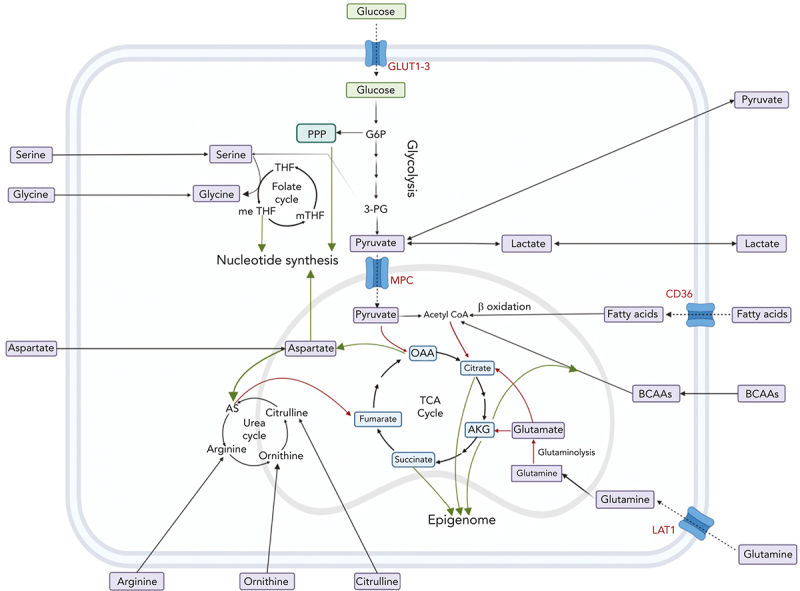

## Teaser

Rattigan and colleagues examine the current understanding of the role of metabolism in proliferation and survival of cancer cells. The authors review the multiple metabolic pathways beyond the “Warburg effect” that impinge on and are modified by leukemia cells. They discuss the degree with which these pathways are shared with normal hematopoietic stem cells and investigate the challenge to discover safe entry points for exploiting metabolic dependence to promote successful therapy without negatively affecting normal hematopoietic stem cells and immune cells.

## Introduction

In 1923, Otto Warburg reported that tumors had high fermentation rates, producing high levels of lactic acid.^1^ The rapid conversion of glucose to lactate in the presence of oxygen has become known as the Warburg effect. This effect, like many other features of cancer, is also present in rapidly proliferating normal cells.^2^ Rapid growth of normal cells is required during gestation, cellular replenishment that takes place after chemotherapy, and expansion of immune cells during infection.^3^^,^^4^ Nevertheless, deregulated metabolism is now a well-established characteristic of cancer.^5^ This deregulated metabolism in cancer is required to supply the macromolecules needed for cell division as well as to meet energetic and redox balance.^6^ Depending on the tissue of origin, cancer may be auxotrophic for certain nutrients, and how they upregulate their metabolism or modify their niche can uniquely expose vulnerabilities that are not present in their normal counterparts.^7^, ^8^, ^9^

Research in cancer metabolism has already yielded results that have been translated to a clinical setting or clinical trials.^10^ In the context of anticancer therapies, a key consideration for targeting a metabolic pathway is whether normal cells are dependent on it. Because upregulated metabolism is a requirement for rapidly proliferating cells, the immune system is an excellent model in which unintended effects on normal cells can be examined. We can also draw parallels between noncancerous diseases involving the immune system (infections and autoimmune diseases). There is also the metabolic interaction between cancer stem cells, immune cells, and the niche to consider because all 3 coexist. Here, we focus on what is known about metabolism in the context of normal hematopoiesis and leukemia, and what the field of hematology can learn from the metabolism of metastasis. We will extend our focus beyond the Warburg effect to give a general overview of the different metabolic strategies normal and cancer stem cells apply.

## Hematopoiesis

**Figure 1.**
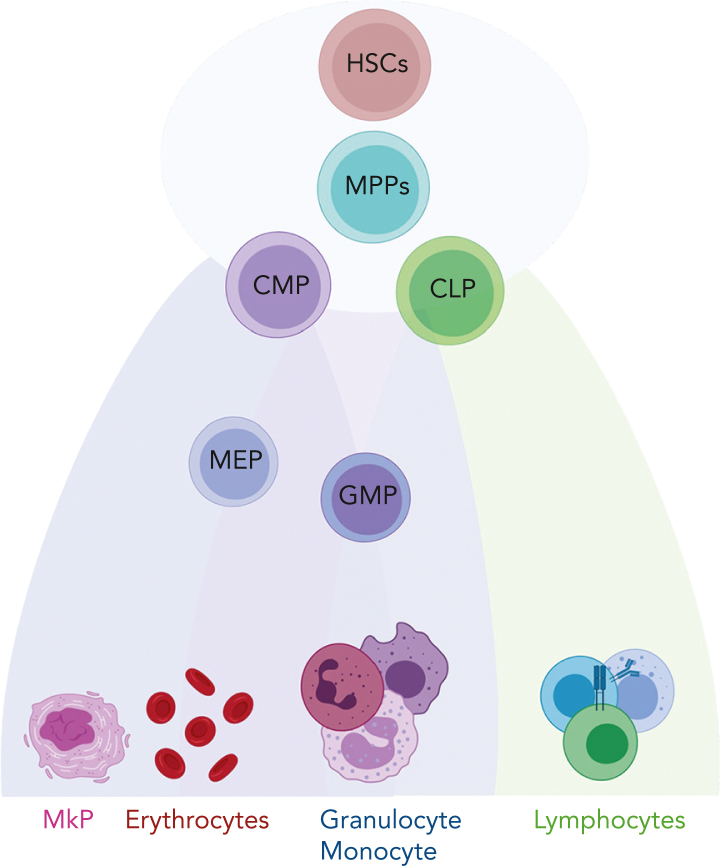
**Schematic representation of hematopoiesis.** In this reconciled model of HSC differentiation, HSCs and their downstream progenies, multipotent progenitor (MPP), common myeloid progenitor (CMP), and common lymphoid progenitor (CLP) cells are part of a continuum and do not represent discrete cell types. They gradually acquire lineage-specific transcriptomic states and differentiate into lineage-restricted cells. GMP, granulocyte-macrophage progenitor; MEP, megakaryocyte-erythroid progenitor; MkP, megakaryocyte progenitor.

The hematopoietic system supports the supply of the majority of mature blood and immune cells, which are derived from multipotent and self-renewing hematopoietic stem cells (HSCs)^11^ (Figure 1). Recent technological advances, including single-cell omics and lineage tracing techniques, have revealed that hematopoiesis is a continuous differentiation process as opposed to the discrete differentiation model, which suggests that hematopoiesis is a stepwise process with a tree-like hierarchy.^12^, ^13^, ^14^, ^15^ In the continuous model, HSCs and their downstream progenies such as multipotent progenitor, common myeloid progenitor, or common lymphoid progenitor cells are part of a continuum of low-primed undifferentiated stem and progenitor cells and do not represent discrete cell types.^13^ This cell continuum exists within the Lin^−^CD34^+^CD38^−^ compartment, whereas discrete populations become established when cells upregulate CD38 and gradually differentiate to lineage-restricted cells.^13^ Although the classical model of hematopoiesis suggests that HSCs are a homogeneous population, recent studies have demonstrated that HSCs differ in their self-renewal ability and lineage-specific outputs.^16^, ^17^, ^18^, ^19^ This heterogeneity is not only a result of cell-intrinsic factors which include, for example, genetic mutations, chromatin architecture, but also extrinsic stimuli such as exposure to the bone marrow (BM) microenvironment.^20^, ^21^, ^22^ Interestingly, heterogeneity in the metabolic activity of HSCs is associated with differential self-renewal and differentiation outputs.^23^^,^^24^ Notably, HSC heterogeneity has particular relevance in myeloid leukemias, in which HSCs and related progenies are the cells of origin. Such preexisting heterogeneity in the HSC compartment might be relevant in both leukemogenesis and therapy response.

## Myeloid leukemia: a model of stem cell–driven cancer

Leukemia is a group of hematological cancers that results from the dysfunctional proliferation of leukocytes. Leukemias are clinically classified into lymphoid and myeloid leukemia according to the type of the predominant proliferating cell and into chronic myeloid leukemia (CML) and acute myeloid leukemia (AML) according to the degree of cell differentiation. Chromosomal rearrangements and genetic alterations arise in hematopoietic stem and progenitor cells, also known as leukemic stem cells (LSCs) and leukemia-initiating cells (LICs).^25^ AML is characterized by complex mutational background making current treatment unable to offer more than moderate success, especially for older patients.^26^ There is evidence of preleukemia driven by early mutations such as DNA methyltransferase 3A, methylcytosine dioxygenases TET1 and TET2, isocitrate dehydrogenase 1 (IDH1) and IDH2, and TP53 (which encodes p53); whereas late mutations such as FMS-like tyrosine kinase-3 (FLT3) or KRAS promote proliferation, block differentiation, and drive the progression of AML.^27^ For example, FLT3 undergoes internal tandem duplication (ITD) to give rise to constitutively active FLT3-ITD. CML originates in a single HSC with a chromosomal translocation t(9;22) that gives rise to the Philadelphia chromosome and the BCR-ABL tyrosine kinase.^28^, ^29^, ^30^ In addition, there is a rare blood cancer called myeloid proliferative neoplasms (MPNs) that is driven by the acquisition of mutation to a tyrosine kinase (JAK2-V617F) in an HSC.^31^

### Current therapies

The introduction of tyrosine kinase inhibitors (TKIs), such as imatinib, that target the BCR-ABL oncoprotein has revolutionized CML therapy.^32^ Similarly, for MPNs, the use of the JAK2 inhibitor ruxolitinib is effective. For AML, chemotherapy known as the 3 + 7 regimen (3 days daunorubicin + 7 days cytarabine) is the most common standard of care.^26^ Inhibitors of FLT3-ITD have been developed and have had a limited clinical effect. Other targeted therapies under evaluation include inhibitors of mutant IDH and apoptosis-mitochondrial protein, BCL-2. Importantly, even with highly effective TKIs available to target BCR-ABL, several studies have shown that CML LSCs can persist in a BCR-ABL–independent manner.^33^^,^^34^ How metabolic rewiring is a characteristic of LSCs that confer TKI resistance will be discussed in the "Role of metabolism in leukemia" section. In addition, we will give examples of similar metabolic reprogramming in lymphoid leukemia and metastatic cancers.

### Identification of HSCs vs LSCs

One of the main technical challenges in studying metabolism in LSCs is that this is a rare cell population that requires enriching using magnetic beads separation and/or flow cytometry; processes that on their own can cause metabolic changes.^35^ Another critical consideration is the definition and functional characterization of stemness. For the latter, the gold standard is long-term engraftment in primary or secondary recipient mice.^36^ The human HSC can be characterized as Lin^−^CD34^+^CD38^−^CD90^+^, whereas its mouse equivalent is Lin^–^c-kit^+^, Sca-1^+^, CD48^−^, CD150^+^ (LSK-SLAM).^37^^,^^38^ Although the CML LSCs can be robustly characterized as Lin^−^CD34^+^CD38^−^CD90^+^CD93^+^;^39^ AML LSCs can phenotypically be CD34^−^CD38^−^, CD34^+^CD38^−^, CD34^+^CD38^+^, or CD34^−^CD38^+^, and sometimes all 4 are present in individual patient samples.^39^ In addition, recent studies have found that AML LSCs are low in reactive oxygen species (ROS) and can be defined by the absence of NKG2D.^36^^,^^40^ This complexity has important implications for study design factors such as mouse model choice and patient stratification and subtype of focus. It is also important to note that studies into hematopoiesis and leukemia often apply a mixture of murine and human models/samples and can differ in the enrichment/purity of stem cells that are studied. This is an important point because modeling niches, such as BM and spleen, necessitate murine models, whereas at the same time, there are intrinsic and extrinsic differences between mice and humans. Information regarding stem cell markers for the studies mentioned in this review is summarized in Table 1.

**Table 1.** **Stemness markers and used functional stem-cell assays**

Murine markers	Normal vs disease	Human markers	Patient-derived xenograft	Single-cell RNA sequencing	References
Lin^−^/LSK					41, 42, 43, 44, 45, 46
Lin^−^/LSK			+		47
LSK-SLAM					38,48, 49, 50, 51, 52, 53, 54, 55, 56
LSK-SLAM	+				57, 58, 59
LSK-SLAM				+	60, 61, 62, 63
LSK-SLAM	+		+		64, 65, 66, 67
LSK-SLAM		CD34^+^/CD38^+^CD38^−^			34,57,68, 69, 70, 71, 72, 73
LSK-SLAM			+		74,75
		CD34^+^/CD38^+^CD38^−^			33,76, 77, 78
		CD34^+^/CD38^+^CD38^−^	+		37,39,79, 80, 81, 82
		CD34^+^/CD38^+^CD38^–^		+	13
			+		83, 84, 85, 86, 87, 88, 89, 90, 91
			+	+	92,93
		ROS-low	+/clinical trial		40,94, 95, 96, 97
		NKG2D^−^	+		36

“Murine markers” refer to markers used to characterize stem cells in murine models. Murine studies were examined in the effect of gene knockout in both normal and disease contexts. “Human markers” refer to markers used to characterize stem cells in human cells. A ‘+’ in patient-derived xenograft column indicates if a patient-derived xenograft model was used in the given study. A ‘+’ in single-cell RNA sequencing indicates if single-cell RNA sequencing was used in the given study.

## The metabolic cost of function: HSCs and immune system

Although the deregulation of metabolic pathways in cancer stem cells can be an attractive therapeutic target, it is important to consider the role of these pathways in nonmalignant tissues. As previously mentioned, hematopoiesis is a constant and energetically-demanding process. However, once hematopoietic cells reach maturity, their role in a functioning immune system requires adaptation to substantial energetic demands. The rapidly expanding immune metabolism field has provided numerous examples of this, in which immune cells require upregulation of multiple metabolic pathways to support rapid expansion and resolution of infection and inflammation. Interestingly, although both normal and malignant cells share common metabolic dependencies, the latter can be more sensitive to metabolic inhibitors.^68^^,^^69^

### Glycolysis and its role in hematopoiesis

**Figure 2.**
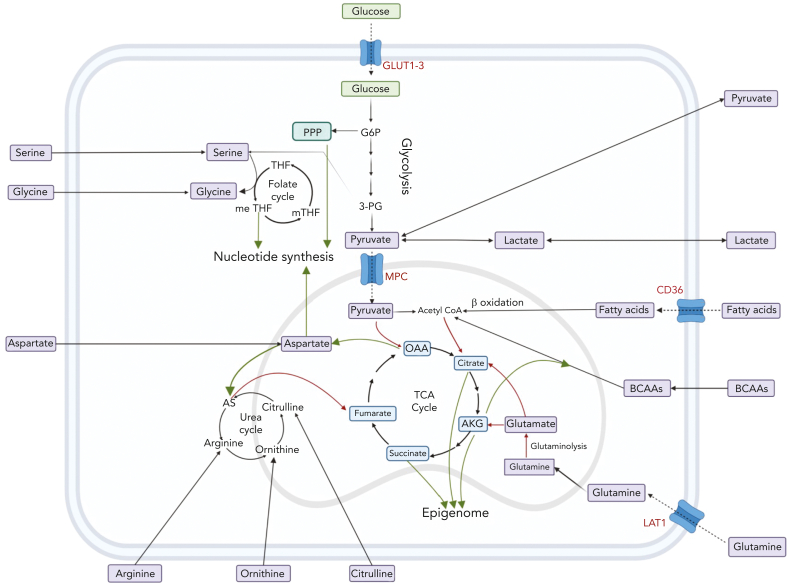
**The interplay between amino acids, carbon substrates, and nonenergetic functions.** Please note for simplicity, polyamine synthesis, mitochondrial folate cycle, and citrate export to cytoplasm for fatty acid synthesis via acetyl-CoA are omitted. Red lines denote reactions that add to central carbon oxidation; green lines denote lines that will divert from central carbon oxidation. 3-PG, 3-phosphoglyceric acid; AS, argininosuccinate; G6P, glucose 6-phosphate; m-THF, methyl-tetrahydrofolate; me THF, methenyl-tetrahydrofolate; OAA, oxaloacetate; THF, tetrahydrofolate.

Although glycolysis is a source of adenosine triphosphate (ATP), it also facilitates the transition of glucose to multiple critical pathways including the pentose phosphate pathway (PPP), which is needed for nucleotide synthesis and reducing equivalent reduced NAD phosphate (Figure 2). The activity of the PPP enzyme, glucose 6-phosphate dehydrogenase, and glutamine import is essential for erythroid commitment.^70^ In contrast, promoting glycolysis via antagonized peroxisome proliferator–activated receptors γ signaling was shown to drive the expansion of human cord blood HSCs and hematopoietic progenitor cells ex vivo.^57^ Notably, such expansion of HSCs was not recapitulated in mouse models.

### TCA cycle: critical for HSCs and T cells

The end product of glycolysis, pyruvate, can be oxidized in the tricarboxylic acid (TCA) cycle to generate reducing equivalents of NADH and FADH_2_ as well as electrons for the electron transfer chain (ETC) to generate ATP (Figure 2). Ansó et al demonstrated the essentiality of the ETC to HSC function.^48^ The authors found that the loss of the ETC complex III subunit Rieske iron-sulfur protein in mouse HSCs resulted in an impaired differentiation that was associated with an increase in 2-hydroxyglutarate levels. Similarly, a study by Yu et al demonstrated that the ablation of PTPMT1, a PTEN-like mitochondrial phosphatase, in HSC in vivo, resulted in hematopoietic failure owing to a differentiation block that was accompanied by reduced mitochondrial respiration.^49^ It has also been shown that HSCs that undergo a high number of cell divisions have dysfunctional mitochondria.^60^ More specifically, HSCs sequester dysfunctional mitochondria during asymmetric division, ultimately leading to a decline in HSC regenerative capacity. During HSC expansion, upregulated TCA activity is critical in generating aspartate that the cells use to supply purines and asparagine.^50^

In the context of T cells, the mitochondrial pyruvate carrier (MPC) has been shown to be critical for the proper development of αβ T-cell development in the thymus.^98^ Using genetic and pharmacological MPC inhibition, Wenes et al showed that MPC activity prevents memory T-cell differentiation in the tumor microenvironment.^99^ In solid tumors, MPC inhibition impaired antitumor function but was of benefit in chimeric antigen receptor T-cell treatment of lymphoid malignancies. Importantly, transient MPC blockade during chimeric antigen receptor T-cell manufacturing enhanced their antitumor efficacy. Here, MPC inhibition promoted lactate oxidation to sustain the antitumor function of cytotoxic T cells. Furthermore, complex II, which is at the interface between the TCA cycle (succinate dehydrogenase) and the ETC is crucial for the proliferation and inflammatory responses of CD4^+^ T cells.^100^

### Fatty acids: essential for HSC function

Fatty acids can be oxidized in the TCA cycle or can be synthesized from citrate that is removed from mitochondria to make acetyl–coenzyme A (CoA) in the cytoplasm. In the context of HSC response to bacterial infection, CD36 drives fatty acid uptake for β-oxidation.^51^ Without this, HSCs were unable to enter the cell cycle, ultimately resulting in increased mortality. With hundreds of thousands of lipid species, the metabolism of fatty acids is extraordinarily complex. A complete overview of fatty acid metabolism is beyond the scope of the review but is nicely covered by Pernes et al.^101^

### Amino acids and their role in immune metabolism

Multiple amino acids have critical roles in immunity.^102^ Here, we focus on those that have critical roles in immunity, immune-related diseases, and leukemia, and thus are of clinical interest. We give a brief overview of current research. For a detailed review, we also suggest Kelly and Pearce.^102^

#### Arginine, fueling wound healing, and T-cell function

Anticancer therapeutics that lower arginine levels are currently being explored in the clinic. However, there is a growing number of studies that show arginine is also required for immune cell function. Although arginine is required for protein synthesis, it is also metabolized to polyamines. Polyamine metabolism generates hypusine, which is an uncommon amino acid that is critical for protein synthesis. In the context of atherosclerosis, lack of polyamine synthesis enzymes arginase 1 and ornithine decarboxylase in macrophages blocks continual efferocytosis and disease resolution.^103^ Puleston et al found that polyamines are also critical for CD4^+^ T helper-cell function.^104^ The authors found that a deficiency in polyamine-derived hypusine disrupted the TCA cycle and histone acetylation. Similarly, Wagner et al found that blocking polyamine metabolism pushed T helper 17 cells toward a regulatory profile (regulatory T cells).^105^ Geiger et al revealed that L-arginine is critical for the metabolic upregulation, survival, and anticancer activity of central memory T cells.^106^ Remarkably, this study found that arginine is bound to multiple proteins, thus changing their confirmation.

#### Glutamine and cysteine: roles in inflammatory disease and anticancer therapy

Blocking glutaminolysis using BPTES or CB839 (telaglenastat) has shown promising results in rheumatoid arthritis,^107^ experimental autoimmune encephalomyelitis,^108^ and Sjögren Syndrome.^109^ Targeting the glutamine-cysteine antiporter has also shown promise in experimental autoimmune encephalomyelitis.^110^ Notably, a cysteine transporter inhibitor, sulfasalazine, is approved to treat rheumatoid arthritis, ulcerative colitis, and Crohn disease. In a recent study, Best et al showed that although glutaminase is upregulated in KRAS-mutated lung cancer, glutaminase inhibition limited CD8^+^ T-cell clone-type expansion and activation.^111^ Importantly, the authors concluded that combining glutaminase inhibition with anti-PD1 treatment would be detrimental to CD8^+^ T-cell function.

#### Asparagine and anticancer immunity

Asparagine is a nonessential amino acid that can also be made de novo from aspartate. Wu et al showed that in CD8^+^ T cells, asparagine binds and causes phosphorylation of lymphocyte-specific protein tyrosine kinase (LCK), resulting in increased LCK activity and T-cell receptor signaling and antitumor responses.^112^

#### Serine: supporting an activated immune system

Serine is a conditionally essential amino acid with multiple functions, including its requirement for the folate cycle which generates nucleotides (Figure 2). Rodriguez et al described that serine metabolism is important for glutathione (GSH) synthesis to support interleukin-1β cytokine production.^113^ They showed that serine deprivation can limit macrophage activation and improve survival in a sepsis model in vivo. Furthermore, it has been shown that serine is required for optimal T-cell proliferation and, ultimately, modulated adaptive immunity.^114^

#### Branched-chain amino acids (BCAAs): a unique dependency on valine

Taya et al found that valine is critical for HSC maintenance both in vitro and in vivo.^71^ Interestingly, this approach could be leveraged to allow for donor HSC engraftment without irradiation.

### Role of metabolism in leukemia

#### Glycolysis: the historical hallmark

The glycolysis-promoting enzyme PFKFB3 was found to be upregulated in TKI-resistant CML and combining its inhibition with TKI reduced disease level in vivo.^52^ Glycolysis has also been shown to have a critical role in precursor B-cell acute lymphoblastic leukemia (B-ALL) LICs.^92^ Morris et al reported that LICs, which were not enriched in primitive cell populations, had higher hypoxic and glycolytic signatures as opposed to bulk blasts that depended on oxidative phosphorylation (OXPHOS).^92^ Similarly, Dasgupta et al showed that in breast cancer, PFKFB4 activity is required for metastasis.^115^ PFKFB4 was found to promote glycolysis and phosphorylation of SRC-3. SRC-3 subsequently increased the transcription of PPP genes, which generates reducing equivalents and nucleotides (Figure 2); and this was required for metastasis to the lung. Interestingly, in the case of diffuse large B-cell lymphoma, low expression of glyceraldehyde-3-phosphate dehydrogenase/high OXPHOS is predictive of a poor response to the standard combined chemotherapy.^116^ Dietary fructose can be catabolized to glycolytic intermediates. Increased fructose consumption via upregulated fructose transporter GLUT5 has been reported in AML cells.^117^ Notably, GLUT5 inhibition synergized with chemotherapy both in vitro and in vivo. This study by Marriott et al is especially pertinent considering the ever-increasing levels of fructose in modern diets.^118^

#### TCA cycle: from cellular energetics to epigenome

Although the Warburg effect is present in many cancers, recently it has become apparent that OXPHOS is also deregulated in certain cancers, and this is particularly the case in leukemia.^119^ Increased mitochondrial biomass and respiration observed in CML and AML LSCs has successfully been targeted at the level of mitochondrial translation using tigecycline with HSCs not being affected.^80^^,^^81^^,^^83^ Here, tigecycline targeted CML and AML cells but not normal cells in vitro and in vivo. However, a recent clinical trial showed that the maximally tolerated dose of tigecycline failed to reach required inhibitory concentrations in patients with AML, likely owing to the half-life of tigecycline being shorter than previously reported for patients without cancer.^120^ Here, the authors proposed that a more stable formulation, that would allow for continuous infusion instead of once daily infusion, could facilitate the attainment of sustained inhibitory concentrations. An alternative approach is to inhibit the ETC, and this approach has culminated in the latest generation ETC inhibitors, such as IACS-01075, which is currently in clinical trials.^84^ However, two recent trials using IACS-01075 were discontinued due to dose-limiting toxicities that included lactic acidosis and neurotoxicity, bringing into question the safety of ETC inhibitors.^121^ In CML, SIRT1 has been found to drive increased OXPHOS in CML LSCs.^64^ One by-product of increased TCA activity in leukemia is increased TCA cycle metabolite fumarate, which has been shown to drive further mitochondrial biogenesis via maleic enzyme 2.^41^ The functional role of increased OXPHOS requires further investigation because AML and CML LSCs have been found to have limited spare respiration capacity and may even consume ATP.^76^^,^^85^

In the case of T-cell ALL (T-ALL), the dependence on pyruvate dehydrogenase (PDH)–oxidized glucose is similar to that of the normal T-cell progenitors. Here, PDH-oxidized pyruvate was shown to be required for GSH synthesis, which highlights the conservation of metabolism within cell lineages despite disease status.^58^ In breast cancer metastasis, AMPK phosphorylation of PDH has been shown to drive increased TCA cycle activity, and this axis correlated with poor patient prognosis.^122^ AMPK has been shown to have a similar role in B-ALL.^86^ Similarly, Bader et al showed that in prostate cancer, upregulation of MPC facilitates upregulated TCA cycle activity which is critical for tumor growth.^123^ Breast cancer cells have been shown to depend on pyruvate from the tumor microenvironment in the context of collagen-based remodeling during lung metastasis^124^ (Figure 2). More specifically, extracellular pyruvate was metabolized to α-ketoglutarate (AKG), which in turn increased collagen prolyl-4-hydroxylase. Prolyl-4-hydroxylase, in turn, led to increased collagen and thus remodeled the extracellular matrix to facilitate metastasis.

OXPHOS has also been shown to be important in the context of therapy remission for both B-ALL and AML.^125^^,^^126^ In AML LSCs, TET3 has been described to drive increased central carbon metabolism.^42^ In homeobox A9–dependent AML, increased jumonji C–containing H3K9 demethylase was found to drive in vivo proliferation and tumorigenesis via upregulated glycolytic and oxidative pathways.^74^

In CML, treatment with BCR-ABL–targeting TKI failed to eradicate LSCs.^34^^,^^127^ Patel et al showed that in CML LSCs, a BCR-ABL–independent STAT3 program resulted in dysregulated mitochondrial metabolism, ultimately leading to TKI-persistent LSCs being dependent on glycolysis and thus, sensitive to glycolytic inhibition.^72^

Inhibitors of BCL-2, such as venetoclax, are showing promise in targeting energy metabolism in AML in the clinic.^94^ Reported escape mechanisms to BCL-2 inhibition in AML are increased fatty acid oxidation that replaced BCAAs as a TCA cycle fuel and nicotinamide metabolism which supplies the TCA cycle with NAD^+^.^95^^,^^96^ Chen et al showed that targeting mitochondrial structure sensitized AML to BCL-2 inhibition.^128^ Similarly, Bosc et al showed that in an in vivo model of chemo-resistant AML, survival of mice could be prolonged by combining OXPHOS inhibition with chemotherapy.^93^ In B-ALL, resistance to BCL-2 inhibition is mediated by increased MCL-2, AMPK activity, and OXPHOS.^129^

The link between metabolism and the epigenome is an exciting and emerging area of research. Jiang et al showed that in MLL-rearranged AML, increased acetyl-CoA prevented BET protein recruitment to chromatin owing to increased histone acetylation, ultimately leading to the upregulation of leukemogenic genes.^47^ The role of TCA cycle–derived acetyl-CoA on chromatic acetylation and the role of TCA cycle–derived succinate, fumarate, and AKG with the folate cycle on chromatin methylation is beyond the scope of this review and is covered in detail by both Intlekofer and Finley and Vetrie et al.^27^^,^^130^

#### Fatty acids: fueling leukemia’s oxidative fire

In addition to contributing to the cell membrane, fatty acids can be oxidized in TCA cycle to generate ATP. Interestingly, fatty acid metabolism can drive venetoclax escape in leukemia.^96^ As mentioned in the context of the leukemic niche, fatty acid metabolism also facilitates cytarabine relapse in AML.^75^ In these studies, increased fatty acid metabolism was driven by increased uptake via CD36 (Figure 2). Furthermore, Savino et al demonstrated that ALL cells upregulate fatty acid synthesis on migration to the central nervous system.^131^ As stated earlier, the literature on fatty acid metabolism is vast and for further reading of the field in the context of cancer we point the reader to an excellent review by Hoy et al.^132^

#### Amino acids, protein synthesis, and beyond

Amino acids have multiple roles beyond protein synthesis with their metabolism exquisitely linked with central carbon metabolism (Figure 2). In addition, cancer can source them from the microenvironment, sparing the cost of synthesis. Here, we focus on the metabolism of amino acids implicated in cancer.

##### Arginine: an auxotrophic-driven vulnerability

Similar to T cells, multiple leukemia types are auxotrophic for arginine, often owing to a lack of argininosuccinate synthase 1. This has been exploited using chemically stabilized (PEGylated) human arginase, which depletes exogenous arginine in vivo.^133^^,^^134^ Arginase and a similarly therapeutic enzyme, arginine deiminase, are currently in clinical trials.^82^ Although the urea cycle can support the TCA cycle via fumarate, 1 advantage of low argininosuccinate synthase 1 is increased aspartate available for nucleotide synthesis^135^ (Figure 2). This may be also the case for rapidly proliferating normal immune cells. It is important to consider that AML samples also release arginase which impedes T-cell function, so the use of arginine-depleting therapeutics will have similar effects.^136^

##### Glutamine and cysteine: the redox defense of cancer

Although the TCA cycle has a major role in cellular energetics, it also produces aspartate that can be used for high rates of nucleotide synthesis necessitated by rapid proliferation (Figure 2). Thus, it is critical to replenish the cycle with more carbons than the 2 supplied from pyruvate via PDH. In this context, glutamine can contribute 5 carbons to the cycle after conversion to AKG. In FLT3-ITD–driven AML, it has been shown that glutamine anaplerosis becomes critical when leukemic cells are treated with a TKI.^87^^,^^137^ Through the use of a CRISPR-Cas9 screen, Gallipoli et al found that inhibiting glutaminase sensitizes AML cells to TKI treatment. The glutaminase inhibitor CB-839 (telaglenastat) has been or is currently in 22 clinical trials for various hematological and solid tumor cancers. In MLL-AF9 leukemia, upregulation of the glutamine transporter ASCT2 is critical for disease progression.^65^ Glutamine and cysteine are required to synthesize GSH, an important antioxidant. Cramer et al generated a PEGylated mutant enzyme with activity and durability sufficient for in vivo cystine and cysteine depletion and used it with great effect in models of leukemia, prostrate, and breast cancer.^138^ Jones et al showed that cysteine depletion resulted in the inhibition of ETC complex II in AML LSCs whereas Badgley et al found that cysteine depletion in pancreatic cancer induced ferroptosis.^97^^,^^139^ Interestingly, Jones et al showed that this was because of the loss of the posttranslational glutathionylation of succinate dehydrogenase, highlighting the complexity of metabolite-protein interactions.^97^, ^139^

##### Asparagine: an early antileukemic target

Although asparaginase that reduces the availability of asparagine is a well-established therapy for ALL,^140^ its effect on CML cells has recently been explored.^77^^,^^141^ Strikingly, these studies confirmed that asparaginase similarly depletes glutamine, also essential for CML cells. Whether there is a synergy from depleting each amino acid is unknown. Notably, in breast cancer models, asparagine only becomes essential when extracellular glutamine levels are decreased.^142^ Pavlova et al found that asparagine was only required to maintain protein translation. Ultimately, cancer cells can lose their vulnerability to chemical inhibition and dietary or enzymatic targeting of these pathways by the upregulation of relevant synthesis pathways.^142^ This has been shown to be the case for T-ALL in which both ATF4 and ZBTB1 transcription factors were recruited to the promoter of asparagine synthase to drive its expression.^143^

##### Serine synthesis vs metabolism

Dietary modulation of serine has successfully been explored in the context of cancer.^144^ Serine is critical for breast cancer metastasis.^145^^,^^146^ Remarkably, high fructose has been shown to increase serine synthesis in AML.^8^ In CML, targeting serine-glycine regulatory enzyme, PRMT7, reprogrammed glycine metabolism, which led to excessive levels of the toxin methylglyoxal and ultimately reduction in the number of CML LSCs.^66^ The role of serine metabolism in hematological malignancies and its targeting using antifolates is a substantial area of research covered in more detail by Zarou et al.^147^

##### BCAAs: key modulators of the epigenome

In both AML and CML, the metabolism of BCAAs has been shown to drive an epigenetic rewiring, that is similar to that caused by 2-hydroxyglutarate.^39^^,^^88^ Although degradation of BCAAs results in acetyl-CoA, a study by Raffel et al found that by converting BCAAs and AKG into BCAAs and glutamate, the transaminase BCAT deprives TET2 of its cofactor for demethylation (Figure 2).^39^ Fascinatingly, the link between BCAAs and epigenetics is bidirectional. Gu et al demonstrated that in normal hemopoiesis, BCAT1 is repressed by EZH2, and loss of EZH2 function leads to increased BCAT1 activity in NRAS-driven leukemia.^59^ The levels of BCAAs in leukemic and normal cells are often controlled by transporter LAT1 ("Amino acid transporters: gatekeepers of amino acid metabolism" section). The role of LAT1 and BCAAs in normal cells is reviewed elsewhere in the study by Kelly and Pearce.^102^

##### Amino acid transporters: gatekeepers of amino acid metabolism

Increased levels of amino acid transporter LAT1 have been shown to be critical for ALL and AML.^67^^,^^148^ LAT1 transports multiple amino acids, including glutamine, into the cell (Figure 2). LAT1 is a heterodimeric protein that comprises SLC7A5 (CD98), which also functions as a vasculature-integrin–binding glycoprotein, and SLC3A2. Notably, in AML, both amino acid and vasculature binding functions of CD98 are required for AML LSC maintenance because partial chimeras were unable to rescue knockout as opposed to the full-length protein.^67^ In addition, cysteine can be targeted via its transporter, using inhibitors, such as erastin, which has shown promise in breast cancer studies.^149^ An overview with key studies describing metabolic vulnerabilities in hematological malignancies is summarized in Table 2.

**Table 2.** **Summary of metabolic vulnerabilities reported in hematological malignancies**

Metabolic pathways/targets	Examples in hematological malignancies	References
Glycolysis	PFKFB3 was found to be upregulated in TKI-resistant CML	52,92,117
	GLUT5 inhibition synergized with standard chemotherapy to eradicate AML cells	
	Glycolytic signatures upregulated in B-ALL	
TCA cycle	Inhibition of OXPHOS/ETC tested in CML and AML	64,80,81,83,125
	Upregulation of OXPHOS confers resistance to B-ALL	
Fatty acid metabolism	Increased uptake of fatty acids confers chemoresistance in AML	96,131
	Upregulation of fatty acid synthesis in the central nervous system ALL	
Amino acids and their transporters	Depletion of exogenous arginine studied in AML and ALL	66,77,82,87,88,133,134,137,141,148
	Inhibition of glutamine anaplerosis sensitizes AML cells to TKI therapy	
	Asparaginase used in the treatment of ALL	
	Targeting of the serine-glycine regulatory enzyme PRMT7 results in the reduction of CML LSCs	
	BCAA metabolism drives epigenetic rewiring in CML and AML	
	Increased expression of amino acid transporter LAT1 in AML and ALL	

## The BM niche

### Impact of the hypoxic BM niche on HSC metabolism

**Figure 3.**
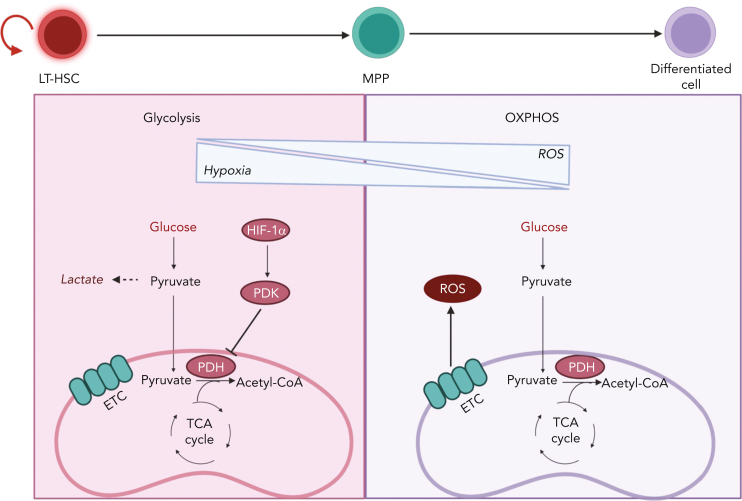
**Transition from glycolysis to OXPHOS during HSC differentiation.** HSCs are known to reside in a hypoxic BM niche and to rely on anaerobic glycolysis. Hypoxic conditions activate HIF-1α, which promotes the activation of PDK. PDK in turns prevents pyruvate entry to the TCA cycle by inhibiting PDH. HSC differentiation is associated with elevation of ROS owing to a metabolic switch toward OXPHOS and increased oxygen consumption. LT-HSC, long-term hematopoietic stem cell.

HSCs reside in a hypoxic BM niche with limited oxygen levels and low levels of ROS (Figure 3). Their position in the BM niche enforces a quiescence state that enables HSCs to respond to emergency situations by exiting from dormancy and initiating the replenishment of the blood system. In the steady state, most HSCs divide on an average once every 30 days.^150^ Cell cycle quiescence not only protects the integrity of adult HSCs via limitation of oxidative stress from increased mitochondrial respiration, but also prevents their exhaustion owing to uncontrolled cell cycle and proliferation.^43^^,^^44^^,^^53^^,^^151^ Thus, HSCs rely on anaerobic glycolysis, whereas elevated ROS increased mitochondrial biogenesis, and increased OXPHOS results in HSC differentiation. If not resolved by the DNA repair machinery, increased production of ROS can result in HSC exhaustion.^23^

Limited oxygen in the BM microenvironment results in the activation of hypoxia-inducible factor 1 (HIF-1), a transcriptional factor essential in the cellular response to oxygen levels and a key player in HSC metabolism (Figure 3). HIF-1 is a heterodimer that is composed of the following 2 components: HIF-1α, whose expression is stabilized by hypoxia; and HIF-1β, which is constitutively expressed. When HIF-1α and HIF-1β associate, they form a transcriptional factor that promotes the expression of multiple glycolytic genes.^23^ Furthermore, HIF-1α promotes the expression of pyruvate dehydrogenase kinase 2 (PDK2) and PDK4, which prevents pyruvate from entering the TCA by inhibiting PDH (Figure 3). The depletion of *HIF-1α* causes an increase in ROS and OXPHOS that ultimately leads to loss of quiescence, although the overexpression of *PDK2* and *PDK4* can rescue such phenotype.^54^

### Metabolic remodeling of the niche

Similar to normal HSCs, LSCs reside in the hypoxic part of the BM. The BM niche facilitates CML LSCs escape from imatinib treatment.^78^ Mechanistically, the hypoxic environment stabilized HIF1-α to allow BCR-ABL–independent survival. Gastel et al reported a transient metabolic vulnerability that emerged in the niche during chemotherapy of AML.^61^ Stromal cells were found to use glutamine to supply AML cells with glutamine-derived aspartate although AML cells use glutamine to fuel pyrimidine and GSH synthesis. The timing of the treatment was critical as targeting pyrimidine metabolism after chemotherapy showed synergy in vivo whereas cotreatment did not. Studying MPNs, Rao et al found that metabolic rewiring driven by mutated JAK2 resulted in hypoglycemia and adipose tissue atrophy.^45^ Here, the authors used pharmacological inhibition of upregulated glycolytic enzyme Pfkfb3 to target the LSC, or separately, a high-fat diet to reverse adipose tissue atrophy.

Using either BCR-ABL or MLL/AF9 fusion models of aggressive acute CML/AML, Ye et al found that AML cells induced high-level production of IGFBP1 from adipose tissue to decrease insulin secretion and sensitivity, and this could be targeted using an anti-IGFBP1 antibody.^62^ The same study also found that the loss of both gut-derived serotonin and microbiota-derived–short-chain fatty acids contributed to perturbed glucose metabolism. This highlights an additional layer of complexity involving the microbiome. For an excellent review on the role of the microbiome in cancer and cancer therapy, refer to the study by Helmink et al.^152^

Multiple studies have shown that in AML, mitochondria are transferred from stromal cells into leukemic cells.^89^^,^^90^^,^^153^ The net result of this is that AML cells are spared the high energetic cost of mitochondrial biogenesis. Finally, adipocytes make up a large percentage of BM in older adults who comprise the vast majority of patients with leukemia, and the adipocyte niche has shown to be advantageous to AML and CML LSCs in terms of maintenance and survival of chemotherapy.^75^^,^^91^

Finally, it is also important to consider that systemic lactate can substantially contribute to the TCA cycle in tumors (Figure 2), which highlights the importance of modeling the circulation of metabolites between multiple organs.^154^

## Direction for the future

It is important to note that the most comprehensive studies into metabolic requirements of either normal cells or cancer cells are those that focus on dependencies, that is, when reviewing the literature, we found much fewer studies showing that a given metabolic pathway is not required. Although the discovery of mutant oncoproteins such as FLT-3 ITD, BCR-ABL, and IDH have offered clinically relevant cancer-specific targets; in most cases, cancer metabolic reprograming involves alterations of pathways that occur in nonmalignant cells. In addition, metabolic reprogramming might persist in an oncoprotein-independent manner, making metabolic rewiring a targetable vulnerability. In prioritizing new metabolic inhibitors, a key consideration is balancing the importance of a metabolic pathway with the efficacy/availability of inhibitors. For instance, 2-deoxyglucose blocks the Warburg effect, yet it has off-target effects and lacks efficacy in the clinic.^10^ Interestingly, there are metabolic inhibitors approved for treating immune disease such as leflunomide (pyrimidine metabolism), currently in trials for multiple cancers, including breast and prostate cancer. In Table 3, we have listed commonly used tool-compound inhibitors or therapeutics that are/have been investigated for anticancer effects in clinical trials. For a comprehensive overview on tool and clinical-grade metabolic inhibitors in the context of cancer- and immune-related disease, we refer the reader to an excellent review by Stine et al.^10^ The similarities between cancer and immune disease metabolism mean that treatments that are found to be effective for one, could be effective for the other, as is the case for methotrexate. However, a major side effect of the high dose of methotrexate, used for hematological malignancies, is BM suppression. To increase the robustness of preclinical studies, normal controls should be used when possible as well as examining if systemic therapy in vivo effects basal complete blood counts or BM composition. We recognize that there are limitations to what can be done in this regard. For example, a study examining a drug effect on LSC burden might not check if the drug effected HSC function (serial transplantation) or immune function (infection or immune therapy). Although there may be limitations to what normal controls can be used for, they are nevertheless particularly pertinent to metabolic inhibitors that are tested in combination with myeloablative agents or immune therapy. Thus, balancing the effect of inhibiting metabolism on cancer stem cells, with that of the immune cells that may be limiting disease progression, or that required for successful immune therapy, remains an open challenge.

**Table 3.** **Summary of metabolic inhibitors currently in clinical trials**

Drug	Pathway/target	Trials (National Institutes of Health unless stated otherwise)
2-Deoxyglucose	Glycolysis	2-Deoxyglucose: 249 trials (majority are for positron emission tomography) lonidamine
IACS-010759, IM156, and CPI-613	TCA/ETC	IACS-010759: 2 trials, IM156: 30 trials, and CPI-613: 30 trials
Mildronate, trimetazidine, perhexiline, and etomoxir	Fatty acid oxidation	Mildronate: 2 trials; trimetazidine: 28 trials, perhexiline: 10 trials, etomoxir: preclinical
BCT-100, AEB1102, and ADI-PEG20	Arginine metabolism	BCT-100: 9 trials, ADI-PEG20: 28 trials, and AEB1102: 3 trials
CB-839 (telaglenastat) and IPN60090	Glutamine metabolism	CB-839: 22 trials and IPN60090: 2 trials
Sulfasalazine, cysteinase, and erastin	Cysteine metabolism	Sulfasalazine: used to treat rheumatoid arthritis, ulcerative colitis, and Crohn disease, 114 trials; cysteinase: preclinical; and erastin: preclinical
Asparaginase: Erwinase or Oncaspar	Asparagine metabolism	Used to treat ALL: 296 trials
Methotrexate and SHIN1/2	Serine metabolism	Methotrexate: used to treat multiple cancer types and autoimmune disease, 2300 trials and SHIN1/2: preclinical
ERG-240	BCAA metabolism	Preclinical
JPH203	LAT1	UMIN000016546

Conflict-of-interest disclosure: The authors declare no competing financial interests.
